# Progress for *Journal of Functional Morphology and Kinesiology* in 2020

**DOI:** 10.3390/jfmk6010011

**Published:** 2021-01-22

**Authors:** Giuseppe Musumeci

**Affiliations:** 1 Department of Biomedical and Biotechnological Sciences, Anatomy, Histology and Movement Sciences Section, School of Medicine, University of Catania, via S. Sofia 87, 95123 Catania, Italy; g.musumeci@unict.it; 2 Research Center on Motor Activities (CRAM), University of Catania, 95123 Catania, Italy; 3 Department of Biology, Sbarro Institute for Cancer Research and Molecular Medicine, College of Science and Technology, Temple University, Philadelphia, PA 19122, USA

## 1. Looking Back on 2020

The *Journal of Functional Morphology and Kinesiology* (JFMK, ISSN: 2411-5142), which was first released in March 2016, developed greatly in 2020. This journal provides an advanced forum for research studies on functional morphology and kinesiology and the regulatory functions of movement. JFMK meets the growing demand for high-quality, peer-reviewed international journals, supplying the easy access and high publicity of open access, the digital object identifier (DOI), ORCID, and CrossRef to all researchers. We are indexed in the Scopus (Elsevier’s abstract and citation database), DOAJ (Directory of Open Access Journals), Scilit (a comprehensive, open-access scholarly database, developed and maintained by MDPI), Google Scholar, World Health Organization, Hinari, FSTA—Food Science and Technology Abstracts (IFIS), and the Norwegian Register for Scientific Journals, Series and Publishers (NSD). Our full texts are archived in CLOCKSS (Digital Archive), e-Helvetica (Swiss National Library Digital Archive), and J-Gate (Informatics India). 

In 2020, JFMK was covered by SCImago Journal Rank with a 1.5 Citescore and reached a Q3-quartiles in the following research fields: anatomy, histology, orthopedics and sports medicine, physical therapy, sports therapy, rehabilitation and rheumatology.

With great pleasure we announce that in early 2021, we will be indexed by PubMed; we have been recently accepted. We hope we can be included in Web of Science in the near future. 

JFMK is a member of the Committee on Publication Ethics (COPE). To verify the originality of content submitted to our journals, we still use iThenticate to check submissions against previous publications. MDPI works with Publons to provide reviewers with credit for their work, and MDPI Scitations Alert to provide our authors information on new publications in their research field. 

The journal publishes articles focusing on molecular, cellular, tissue, system and whole-body responses to a broad range of physical activities. Furthermore, it provides an advanced forum for the analysis of the structure, function, development, and evolution of the cells and tissues of the musculoskeletal system and associated disorders.

We are proud to let you know that, thanks to your continuous support, *Journal of Functional Morphology and Kinesiology* has continued to grow in functional morphology and kinesiology research dealing with the analysis of the structure, function, development and evolution of cells and tissues of the musculoskeletal system, and the whole body. It is my pleasure to confirm the progress recorded in the last few years [1,2,3], as stated in our statistics (https://www.mdpi.com/journal/jfmk/stats). 

Indeed, the number of published manuscripts has jumped from 64 in the 2019 volume to 101 (including editorials) in the 2020 volume, with an increase of more than 37% compared with last year. We rejected 39.73% (67 papers) of the contributions to maintain the high standards of our journal. The *Journal of Functional Morphology and Kinesiology* receives more manuscripts than it is able to publish, and the decision as to which papers are accepted or rejected is a difficult one. The decision is based on several factors, including originality, experimental design, scientific quality, data interpretation, clarity, and English quality, to maintain the high standards of our journal.

In 2020, different Special Issues were completed thanks to the huge support of our editors. They include the following: “Role of Exercises in Musculoskeletal Disorders—3rd Edition”, edited by Prof. Dr. Giuseppe Musumeci and Dr. Silvia Ravalli [4]; “Electromyographic Diagnosis and Rehabilitative Treatment of Focal Neuropathies”, edited by Prof. Dr. Michele Vecchio [5]; “Eccentric Exercise: Adaptations and Applications for Health and Performance—2nd Edition”, edited by Dr. Nicholas Gill, Mr Conor McNeill, Dr. C. Martyn Beaven and Dr. Daniel T. McMaster [6]; “Muscular Strength and Its Influence on Performance Outcomes”, edited by Prof. Dr. Timothy J. Suchomel and Dr. Lachlan P. James [7]; “Psychology of Development and Education Applied to Movement—2nd Edition”, edited by Dr. Marianna Alesi [8]; “Exercise Evaluation and Prescription”, edited by Dr. Cristina Cortis, Dr. Andrea Fusco and Dr. Carl Foster [9]; “Resistance Training for Performance and Health 2020-2021”, edited by Prof. Dr. Antonio Paoli [10]; “Physical Activity Improves Muscle-Cognitive Performance: Impact in Quality of Life”, edited by Prof. Dr. Virginia Tancredi and Prof. Dr. Agata Grazia D’Amico [11]; “Histo-immunology in Exercise”, edited by Dr. Michelino Di Ros [12]; “Oral and Maxillo-Facial Rehabilitation: From Conventional and Digital Diagnostic to Surgeries and Prosthetics”, edited by Luca Fiorillo [13]; “Applied Sport Physiology and Performance—2nd Edition”, edited by Dr. William Guyton Hornsby III [14]; “Knee Kinematics after Total Knee Arthroplasty (TKA): Influence of the Alignment”, edited by Dr. Pier Francesco Indelli [15]; “Health and Performance through Sports at All Ages”, edited by Dr. Gianpiero Greco [16].

In 2020, six distinguished scientists joined the Editorial Board: Prof. Dr. Agata Grazia D’Amico (San Raffaele Open University of Rome, Rome, Italy), Prof. Dr. Virginia Tancredi (University of Rome Tor Vergata, Rome, Italy), Dr. Dawson J. Kidgell (Monash University, Australia), Prof. Dr. Jose Antonio (Nova Southeastern University, Davie Florida, USA), Prof. Dr. Pasqualina Buono (Università Parthenope, Naples, Italy), Prof. Dr. Douglas Powell (University of Memphisdisabled, Memphis, USA) and Dr. Adam Wells (University of Central Florida, FL, USA), for a total of 79 editorial board members, seven advisory board members, and the editor-in-chief.

All articles published in the *Journal of Functional Morphology and Kinesiology* will be published in full open access, and in order to provide free access to readers, and to cover the costs of peer review, copyediting, typesetting, long-term archiving, and journal management, an article processing charge (APC) of 1400 CHF (Swiss Francs) will be applied to papers accepted after peer review. 

## 2. Looking Forward to 2021

In 2021, we shall continue our efforts to improve the journal through further growth and increased visibility. 

In order to achieve this target and lay a strong foundation for publications in 2021, and in our application for indexing, we have made the following plans:

- Follow up the planned papers from editorial board members and agreed authors;
- Contact international conferences recommended by the editor-in-chief or by editorial board members, and try to establish media partnerships with them to make JFMK increasingly well-known among scholars;
- Communicate with editorial board members regularly and ask for their help and suggestions for journal development;
- Post high-quality papers through social media (e.g., LinkedIn, Twitter, and Facebook) and increase online readership;
- Reduce the processing time of each submitted manuscript;
- Try to have publications indexed by the Emerging Sources Citation Index (Web of Science), by EMBASE (Elsevier) and by Web of Science—Clarivate;
- Try to improve the Citescore in the SCImago Journal Rank in the kinesiology-related sections, such as anatomy, histology, orthopedics and sports medicine, physical therapy, sports therapy, rehabilitation and rheumatology;
- Try to be reach the First Impact Factor released by Clarivate Analytics;
- Accomplish, for our authors, the best JFMK paper award and the JFMK travel grant award;
- Garner, for the sake of journal promotion, support from sponsors for our editors to participate in, and disseminate our journal to, international conferences.

From 2021, MDPI will include the accepting Academic Editor’s name on published articles, once they have accepted that manuscript after full peer review. This establishes greater transparency for the readership, demonstrates the care that our Academic Editors take in making decisions, and offers full acknowledgement of the effort put in when making expert judgements about the suitability of a manuscript for publication. We strongly believe that this will also support quality during peer reviews.

We hope that you share our enthusiasm for this journal, and we look forward to working with you to make JFMK a leader in its field. Your contributions are vital for the success of this new journal. We look forward to receiving your contributions (papers, reviews, etc.), and proposals for Special Issues are always welcome.

I have personally found this to be quite a challenge, not helped by COVID-19, but more due to the special position that JFMK is trying to negotiate in the highly competitive publishing landscape. I wish you a healthy and prosperous new year, and look forward to continuing to expand the reach and impact of the journal with your help next year.

I also take this opportunity to warmly thank, for their confidence, the following: our authors, readers, and reviewers; our editorial advisors; eminent scientists in these fields who, with their experience and important suggestions, guide us in this great enterprise; our excellent editorial board members whose depth of experience covers a very broad spectrum of different disciplines related to the morphology and kinesiology arenas; the managing editor Ms. Molly Lu for her huge support, the publishing manager Dr. Unai Vicario and the other members of the Editorial office who, day after day, thanks to their valuable contributions, ensure the growth of this journal; and, finally, all members of our teams in Basel, Barcelona, Beijing, Belgrade, Romania, Tokyo, and Wuhan, as well as our sponsors.
